# Meditation-Induced Self-Boundary Flexibility and Prosociality: A MEG and Behavioral Measures Study

**DOI:** 10.3390/brainsci14121181

**Published:** 2024-11-26

**Authors:** Yoav Schweitzer, Fynn-Mathis Trautwein, Yair Dor-Ziderman, Ohad Nave, Jonathan David, Stephen Fulder, Aviva Berkovich-Ohana

**Affiliations:** 1 Edmond Safra Brain Research Center, Faculty of Education, University of Haifa, Haifa 3498838, Israel; schweitzeryoa@telhai.ac.il (Y.S.); yairem@gmail.com (Y.D.-Z.); yonidavid9@gmail.com (J.D.); 2 The Integrated Brain and Behavior Research Center (IBBRC), University of Haifa, Haifa 3498838, Israel; 3 Department of Counseling and Human Development, Faculty of Education, University of Haifa, Haifa 3498838, Israel; naveohad@gmail.com; 4 Department of Psychology and Social Work, Tel-Hai Academic College, Qiryat Shemona 1220800, Israel; 5 Department of Psychosomatic Medicine and Psychotherapy, Faculty of Medicine, University of Freiburg, 79085 Freiburg im Breisgau, Germany; 6 Department of Cognitive Sciences, Hebrew University of Jerusalem, Jerusalem 9190501, Israel; 7 The Israel Insight Society (Tovana), Kibbutz Ein-Dor, R.D. Izrael 1933500, Israel; stephenfulder@gmail.com; 8 Department of Learning and Instructional Sciences, Faculty of Education, University of Haifa, Haifa 3498838, Israel

**Keywords:** self-boundary (SB) flexibility, prosociality, meditation

## Abstract

Background: In the last decade, empirical studies on the beneficial effects of meditation on prosocial capacities have accumulated, but the underlying mechanisms remain unclear. Buddhist sources state that liberating oneself from a fixed view of the self by gaining access to its transitory and malleable nature leads to increased compassion and other prosocial traits. These, however, have not yet been empirically tested. Methods: The current study aims at filling this gap by first examining whether 44 long term meditators differ from 53 controls in prosocial capacities on different levels of the socio-cognitive hierarchy, and second by examining whether these are associated with meditation-induced ‘selfless’ states, operationalized here as the sense of boundary (SB) flexibility. We capitalize on our previous work on the neurophenomenology of mindfulness-induced SB dissolution, which yielded a neural index of SB-flexibility, solely for the meditators, and examine its correlations with a battery of validated behavioral prosociality tasks. Results: Our findings reveal enhanced low-level prosocial processes in meditators, including enhanced emotion recognition and reduced outgroup bias. We show the stability of SB flexibility over a year, demonstrating consistent high beta deactivation. The neural index of SB flexibility negatively correlates with recognizing negative emotions, suggesting a link to reduced social threat perception. Conclusions: These results connect the neural correlates of SB flexibility to prosociality, supported by stable high beta deactivations. We expect the results to raise awareness regarding the prosocial potential of flexing one’s self-boundaries through meditation.

## 1. Introduction

Contemplative practice in the Buddhist tradition aims to cultivate insight concerning the “illusory” nature of the self by experiencing the self as a transitory process rather than a rigidly reified and centralized entity [1]. This insight experience is expressed in Buddhist writings as the soteriological doctrine of anatta, or “no-self” [2] (p. 2), and is a core teaching of all Buddhist practice traditions. Dedicated Vipassanā practice, stemming from Theravada Buddhism, is designed to lead, over time, to states of such insights. This shift in self-experience, characterized as a reduction in self-reification [3] and self-centeredness and an increase in the fluidity and interconnectedness of the self [4], is said to lead to increases in compassion and prosocial orientation toward others [5,6]. While increasing evidence supports the general effect of meditation practice on prosociality [7,8,9], and the study of the “self” construct in the context of mindfulness meditation has gained prominence [10,11,12], the mediating role of a change in the structure of the self has so far not been backed by empirical evidence.

One of the suggested candidates is “selfless” or “self-transcending” experiences [13,14,15]—experiences of the self as lacking reification and fundamentally interconnected with others. This line of thought has also been suggested in studies tying prosocial outcomes of meditation to Buddhist acknowledgment of emptiness and selflessness [2,16], as well as to self-transcendent [17], non-dual [18], and mystical experiences more generally [19]. This paper aims to examine the link between mindfulness meditation and prosocial behavior in relation to an altered sense of self, specifically in relation to selflessness states.

In the last decade, the blurring of the distinction between self and others, measured and manipulated via various techniques, has been linked to changes in neural and behavioral signatures of social processing [20,21]. Among these, virtual-reality manipulations implementing multisensory stimulation to attenuate self–other distinctions (e.g., the enfacement illusion) were found to reduce social prejudice [7,22,23] as well as increase prosocial affect and increase emotion recognition [23,24].

One suggested explanation is that bodily and affective states of self and other map onto a shared representational space, providing a basis for action, affect sharing, and intention understanding [25]. This is supported by evidence showing that both social processing and manipulation of self–other boundaries involve activation of similar brain regions, including the posterior cingulate cortex (PCC) and temporoparietal junction (TPJ) [20,26,27]. These two regions are suggested to support multisensory integration [28], which is at the core of self–other differentiation, hence enabling one to sense and maintain one’s sense of self-boundaries (SB) [29,30].

While the benefits of blurred SB states for enhanced social processing have received substantial support [27,31,32], there are cases in which losing the self–other distinction may bear possible risks [33], ranging from empathic distress [34] through mental health deterioration [35,36] and even to psychotic experiences [37].

Disturbances in SB are common in a wide range of psychopathologies. For instance, schizophrenia [38] and post-trauma are characterized by an involuntary shift from an ordinary to a rigid and closed SB, possibly enacted as a defense mechanism [39,40]. Similarly, elevated levels of social anxiety have been tied to a need for greater interpersonal space [41], and abnormalities in personal space regulation have been reported in schizophrenia [42], autism [43], and abnormal attachment patterns [44]. In addition, depersonalization disorder is characterized by a sense of unreality about the self and the world, reflecting a disturbed SB mode [45]. Thus, it is important to study the conditions under which SB alterations are safe and manifest as wholesome experiences in both the personal and interpersonal domains, as the two are evidently intertwined.

Based on phenomenological studies, Ataria [46] has argued that the SBs are critical for mental health, and too rigid, as well as too weak boundaries, can both have harmful consequences. This stance has received support from recent SB-related psychopathology studies [47,48] and theoretical accounts [49,50]. Hence, prosocial behavior requires a healthy balance between self–other blurring and self–other distinction [25], which may bear therapeutic implications for clinical and non-clinical populations. The working hypothesis of the current study is that the ability to volitionally modulate the permeability of one’s SB (in the following referred to as SB flexibility) reflects such a healthy balance and thus will be associated with enhanced prosociality. We test this notion in a sample of experienced meditators due to their proven capacity at skillfully manipulating their SB, as subsequently detailed.

This line of work is largely within the field of studying meditation-induced alterations to the sense of self. The notion of sense of self is grossly divided by cognitive philosophers into two concepts [51]: the narrative-self (conceptual, autobiographic identity with continuity across time) and the minimal-self (a momentary, perceptual, and embodied self). These two notions of self-constitution find confirmation in cognitive neuroscience, being associated with distinct brain regions, frequencies, and networks. It is widely accepted that the default mode network (DMN) supports the narrative-self, or self-related processing [52]. Numerous studies have documented the effect of meditation on narrative modes of self-processing by measuring changes in DMN activity, both as a state and trait (e.g., [53,54,55], reviewed by [56]). However, studies on meditation-related alterations to the minimal-self are less frequent [51,57], and the processes leading to selflessness state experiences and how they can transform into a trait have only recently been suggested by Berkovich-Ohana and colleagues [58].

**Figure 1:**
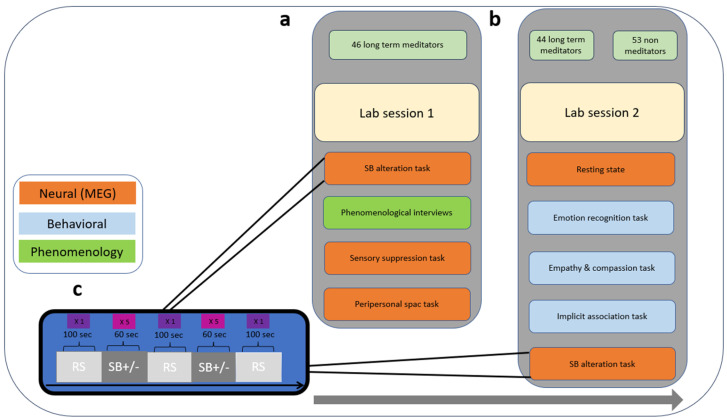
Lab sessions 1 and 2 design: (**a**) Session 1 included neural measurements (orange) and phenomenology interview (green). (**b**) Session 2 included neural measurements (orange), as well as behavioral measurements (blue). (**c**) The order of the main task (self-boundary—SB), including three resting states (RS, pale gray) and ten counterbalanced SB maintenance (SB+) and SB dissolution (SB−) epochs (dark gray) in between. The grey arrow indicates the timeline for a and b.

Against this background, a series of studies from our lab have established that long-term meditation practitioners are able to volitionally attenuate their minimal self, operationalized as their sense of self-boundaries (SB) under rigorous lab settings (reviewed by [59]). A recent study tying the phenomenon of selfless experiences to an embodied continuum [60] supports this operationalization. In this set of studies, we implemented neurophenomenology, a scientific research program aimed at bridging third- and first-person approaches to studying the human mind [61]. We combined phenomenological interviews with magnetoencephalography (MEG) in collaboration with long-term meditators. The preliminary proof-of-concept study [62] demonstrated that a small group (n = 12) of proficient practitioners were able, on demand, to demonstrate a volitional phenomenological transition between narrative self, minimal self, and a selflessness state in a lab. The main findings have shown that minimal-self-attenuation is characterized by prominent beta power reductions in PCC/Precuneus and TPJ. The follow-up case study, with a uniquely qualified meditation expert [63,64], zoomed in on the phenomenology of these changes in the SB, showing that it is associated with a reduced experience of ownership, agency, and first-person perspective [63], while the underlying neural correlates were replicated as reduced beta (peaking at 27 Hz) activity within TPJ and PCC/Precuneus. These pilot studies led to a large-scale study (n = 46), with the first aim to replicate and broaden previous results by using meditation state as an active control instead of using a classic resting state (as meditation might linger into resting state [65]) to better understand the neural underpinning of SB dissolution and to see whether this training-induced neural flexibility was maintained over time. We have already published phenomenological results indicating that participants could generate SB dissolution experiences to different degrees of depth, using different attentional strategies, and with variable emotional undertones (for more information, see [66]). We have also replicated our previous neural findings by showing that functional reduction in high beta in PCC underpins the volitional attenuation of SB [67]. Here, we capitalize on this large-scale study, focusing on prosocial processing. The same samples were invited again to the lab a year after the original experiment and repeated the SB MEG task (see Section 2.3.1 and Figure 1a,b), as well as a series of validated questionnaires and prosocial tasks. Additionally, we recruited a new sample of age and gender-matched meditation-naïve participants as a control group (n = 52) who completed the prosociality behavioral tasks only.

Accordingly, the main objective of the present article is to examine the possible link between SB flexibility and prosociality, thus providing initial evidence towards SB flexibility as a candidate mechanism for increased prosociality. Due to the lack of studies on this, prosociality was operationalized using a wide net of tasks gauging it at different levels of the cognitive hierarchy. Our approach is to examine, first, whether meditators perform better on these tasks than controls, and second, whether the meditators’ degree of SB flexibility is associated with enhanced prosociality. Controls did not perform the SB flexibility tasks as these require extended meditative expertise and specific training. As a secondary objective, we also examined whether the neural SB flexibility measures changed from the first to the second lab visit to study long-term effects, as well as verified that SB flexibility was not associated with psychopathology.

Our preregistered (http://doi.org/10.17605/OSF.IO/2P3DG, accessed on 30 August 2021) hypotheses were that meditators would evidence enhanced prosocial traits relative to controls. Specifically, relative to controls, we predicted that meditators would exhibit enhanced emotion recognition indices (1a), enhanced empathy and compassion indices (1b), and reduced ingroup bias indices (1c). Additionally, we hypothesized correlations within the meditators group, such that phenomenological and neural SB flexibility indices would be positively correlated with emotion recognition (2a), positively correlated with empathy and compassion (2b), and negatively correlated with ingroup bias (2c). Finally, we added a non-preregistered exploratory analysis by comparing the neural SB flexibility measures from two independent sessions taking place almost a year later, aiming at testing the stability of this neural measure over time.

## 2. Materials and Methods

### 2.1. Participants

In the first session, the meditation group included a total of forty-six practitioners (aged 26–72, mean age = 39.8 ± 10.9, 27 females and 19 males, all Caucasian) with a wide variance in meditation expertise (115–24,837 h, mean = 3832 ± 4845). Participants were recruited through Tovana (The Israel Insight Society, teaching Theravada Vipassana) in a convenience sample with the criteria of participation in at least one meditation retreat and a minimum of 1 year of practice. Exclusion criteria consisted of conditions that limit MEG data quality (dental splints, artificial cardiac pacemakers), active psychiatric disorders or medication, and not having normal or corrected-to-normal vision and hearing.

For the second session, one year later, thirty-three participants (aged 26–72, mean age = 39.6 ± 11, 22 females and 14 males, all Caucasian) completed the second MEG session, as well as the behavioral tasks in the lab, while eleven participants who could not arrive at the second MEG lab session (Session 2) due to COVID-19 completed the behavioral tasks (without the neural scanning) outside the MEG lab. Hence, we have forty-four participants for the behavioral measures. The control group included fifty-three participants with similar characteristics (aged 25–63, mean age = 36.52 ± 7.7, 24 females and 29 males, all Caucasian) with no meditation practice and with the same exclusion criteria as the meditators. Due to language difficulties and inability to correctly perform the behavioral tasks, one participant was excluded from this group (n = 52). Between subjects, *t*-tests verified that the groups evidenced no age differences (*t*(2, 97) = 1.747, *p* = 0.092), and a chi-squared test verified there were no gender differences (1, n = 99) = 1.8, *p* > 0.05).

Following that, we had n = 96 (44 meditators and 52 controls) for hypotheses 1a–c. This is according to our preregistered power analysis, which suggested a sample size of n = 82 (2 × 41) for addressing hypotheses 1a–c, allowing a power of 0.85 for a 1-sided between-subjects *t*-test at α = 0.05 and a medium 0.6 effect size. However, the dropout for the second lab visit was larger than expected due to COVID-19, and we ended up with a meditation group of n = 33 (23.9%), which is smaller than the original preregistered power analysis for hypotheses 2a–c (n = 42, which allows a power = 0.85 for a 1-sided correlation analysis at α = 0.05 and a medium 0.4 effect size).

All participants signed an informed consent form and were compensated for their time. This study was approved by the ethical IRB of the Faculty of Education, University of Haifa, in accordance with relevant guidelines and regulations for studies with humans.

### 2.2. Study Design and Procedure

A novel design with a mixed-methods approach was implemented, incorporating first- and third-person methodologies as part of a larger project. This study included two experimental sessions set about one year apart (Figure 1a,b).

Prior to these sessions, the meditators group completed a 3-week training aimed at familiarization with the concept and experience of SB dissolution (for details, see [66]). Subsequently, demographic data and self-report questionnaires were collected online. Meditators were invited twice to the lab, with 6–12 months between both visits. In the first session (Figure 1a), meditators completed several MEG measurements. This included a simple SB state alteration task (Figure 1c, referred to as SB alteration task in the following section) used for computation of the neural flexibility index (SBn, see below), as well as an action-based sensory suppression task and a task measuring multisensory integration in peripersonal space (results are reported elsewhere; for details about these tasks, please see the OSF project page: http://doi.org/10.17605/OSF.IO/BSXUA, accessed on 20 December 2018). Immediately after the MEG session, participants completed phenomenological interviews to characterize their experience during the SB state alteration task (for details about the interviews and full results, see [66]).

In the second experimental session (Figure 1b), resting state was recorded, as well as the SB alteration task (to examine our exploratory hypothesis c and for the calculation of SBn flexibility) and several behavioral social processing tasks, including an emotion recognition test (ERT), the socio-affective video task (SoVT), and an implicit association task of ingroup bias (IAT), all of which are described in detail in the behavioral measures Section 2.3.2. The control group completed the second session only, excluding the SB alteration task. Finally, to rule out associations between the SB flexibility training and derivative measures to psychopathology, the participants’ mental health was assessed using a number of questionnaires gauging stress, PTSD, and depersonalization [38,41,44,68].

### 2.3. Measures

#### 2.3.1. SB Alteration Task

The SB alteration task included two consecutive blocks of volitional SB alterations. At the beginning of each block, 45 s of preparation to get into the meditative state were given, followed by 10 epochs of SB alteration (1 min each), where participants were asked to alternate between a clear sense of SB, referred to as SB maintenance, and to dissolve the SB, referred to as SB dissolution. These two conditions were signaled by audio instructions and applied in a counterbalanced order within and across participants. Additionally, a short resting state (90 s) was recorded at the beginning, after five states, and at the end. The non-meditative resting state was used as a control condition, a common practice in meditation research. However, comparisons between meditation and rest are often non-specific, differing in multiple aspects beyond the phenomenology of interest, and are poorly defined—especially in long-term meditators, where meditative characteristics may persist into resting states [65]. To address this, we also included an active control condition in which participants were instructed to meditate while focusing on maintaining a clear sense of boundaries.

Computation of the neural SB flexibility index (SBn): Data recorded during the SB alteration task were used to obtain a neural measure of the ability to volitionally alter the SB. As presented in a separate publication [67], tests in sensor space of the SB alteration task during the first lab session revealed power reductions in the high beta band (peaking at 27 Hz) to be specific to the state of SB dissolution (i.e., reductions were observed for both contrasts, comparing SB dissolution to rest and to SB maintenance). Beamformer-based source imaging was used to project spectral power at this frequency to the level of neural sources, which yielded power reductions in the medial and lateral parietal, as well as somatomotor, regions. In session 2, we had a smaller group (n = 33) of the same cohort who underwent the first lab session. The analysis of session 2 data was identical to session 1’s, again revealing high beta decreases around 27 Hz in parietal clusters (sensor level results in Section 3 and their analyses explained below in Section 2.4.2). Then, the region of interest specified by the overlap of significant voxels from sessions 1 and 2 (source level analyses described in Section 2.4.3) served to calculate the grand averaged power estimates at 27 Hz in the two meditative conditions (SB maintenance and dissolution), as well as a resting state, to assess changes over time (see Section 3.2 for details). As no differences were found between the neural indices of sessions 1 and 2 on both sensor and source levels, power estimates for each condition (SB maintenance and SB dissolution and resting state) in each session (sessions 1 and 2) were extracted by using the overlap mask, thus reflecting the neural beta activity magnitude within the corresponding voxels. The SBn flexibility difference score (SB maintenance minus SB dissolution) was calculated as an average of the extracted data scores from session 1 and session 2. Larger scores in SBn thus reflect larger beta power reductions in SB dissolution vs. SB maintenance, providing a neural proxy of the strength of volitional SB modulation.

Construction of the phenomenological SB flexibility index (SBp): The interviews were inspired by the micro-phenomenological method [69] and were used to inform the neural analysis and characterize meditative states (for details, see [66]). The analysis yielded seven phenomenological categories, where six of them mapped the SB experience, and one category described the different techniques meditators employed for entering the SB states. The six categories were as follows: first-person perspective, self-location, sense of agency, attentional disposition, affective valence, and body sensations. The meditative experiences of each participant were characterized according to these categories by two independent raters. Based on the apparent ordering of subcategories emerging from the qualitative analysis, numeric scores were derived for each subcategory, thereby transforming the categorical data into ordinal dimensions. This allowed the assessment of interrelations between categories, evidencing high intercorrelations between all experiential categories (all r > 0.66) except the valence category (r = 0.33). This intercorrelation was also reflected by the fact that the first component in a PCA analysis accounted for 58% of the variance. Thus, based on these five interrelated dimensions, we constructed an unweighted summary score reflecting the overall depth of dissolution, yielding the final SBp score (for details on this procedure and results, see [66]).

#### 2.3.2. Behavioral Measures

Emotion recognition task (ERT, [70]): The ERT is a behavioral task measuring the recognition of emotions as a trait. Stimuli are based on colored pictures from actors mimicking emotional expressions. The task is constructed of morphed facial emotional expressions from thirteen actors (five males and seven females) and six emotions (anger, disgust, fear, happiness, sadness, and surprise). Each emotion is shown in a frontal view. In this task, 24 randomly ordered trials of the test are presented. Each trial starts with a neutral face that morphed into a fully expressive face. Participants are asked to label the emotion they perceived as fast and as accurately as possible. Accordingly, only trials with successful emotion identification were inserted into the final analysis, resulting in two scores: (1) averaged response time and (2) averaged accuracy scores.

Socio-affective video task (SoVT, [71]): The SoVT measures the empathy and compassion of participants while watching short real-life videos. In this version, 24 short videos, around 15 s each, were shown. The videos were grouped into two conditions: those presenting highly negative emotions and those that presented more neutral, low-emotion situations. After each video, participants were asked to rate the degree of experienced compassion and negative affect (on a 0 to 10 scale). The measure of compassion was defined as the mean compassion rating over all videos in the negative emotion condition, and an index of empathy (in terms of shared negative affect) was computed by subtracting negative affect in the neutral condition from negative affect in the negative emotion condition.

Implicit association task (IAT, [72]): An Arab–Jew version of the IAT was used as an implicit behavioral measure of ingroup (Jews) hidden prejudices towards outgroup (Arab) members [73]. In this IAT version, participants were asked to pair Arab/Jew names and positive/negative traits words into 4 categories: either Arab names/good words vs. Israeli names/bad words or Arab names/bad words vs. Israeli names/good words. These categories consisted of Arab–Muslim names (Ahmed, Samir, Abas, Rafik, Alian), Israeli–Jewish names (Yair, Ronen, Yuval, Dany, Avi), positive trait words (good, clever, clean, nice, strong), and negative trait words (mean, stupid, dirty, evil, weak). The words and names were presented in the center of the screen in random order, and subjects were required to sort them as quickly and accurately as possible by pressing one of two keys (“X” and “M”) on a computer keyboard. We followed the procedure by Greenwald et al. [73] using 40 trials for the combined blocks in which Arab names and positive/negative traits shared a response key, and Jewish names and negative/positive traits shared the other response key, all in a counterbalanced order across participants. Each combined block was preceded by 20 training trials. Negative attitudes to the outgroup were measured as percent differences in reaction time (RT) between pro-Israeli (Israeli/good vs. Arab/bad) and pro-Arab (Israeli/bad vs. Arab/good) category pairings, following Greenwald et al. [73] with a small adaptation according to Bruneau and Saxe [72] (who used the Jew–Arab version as well): IAT bias score = 100 × (Average Pro Israeli RT − Average Pro Arab RT)/(Average Overall RT). A positive score indicates ingroup favoritism, and a negative score corresponds to outgroup favoritism.

#### 2.3.3. Self-Report Questionnaires

Anxiety was measured with the State-Trait Anxiety Inventory trait measure (STAI, [74]. The STAI is a widely used and validated tool for assessing general anxiety levels over time in both clinical and research settings. It consists of 20 self-report items, each rated on a 1–4 Likert scale, with higher scores indicating higher levels of anxiety. Scores are summed to obtain a total score (ranging from 20–80). We used a Hebrew version of the STAI questionnaire. The resulting Cronbach’s alpha coefficient was 0.91, well within the range of the original published scale [74].

Depersonalization was measured via the Cambridge Depersonalization Scale (CDS, [68]). The CDS includes 29 self-report items rated on two Likert scales for frequency (1–4, ranging from never to all the time) and duration (0–10, ranging from a few seconds to more than a week) of experience. The total score on the CDS is calculated by summing the scores for all items in their respective subscales for frequency and duration, each of which yields a separate score. Higher scores on either subscale indicate greater degrees of depersonalization. The CDC was translated from its English version to Hebrew by three bilingual, independent raters. The resulting Cronbach’s alpha coefficient was 0.87.

Post-trauma symptoms were measured using the PTSD Checklist—Civilian Version (PCL-C) [75], which assesses PTSD symptom severity. The PCL-C is a 17-item scale with a 5-point scale, ranging from “not at all” to “extremely” [75]. Participants are asked to report on the frequency of experienced symptoms during the past month and can gain a total score from 17 to 85. In addition to the total score, it can also be used to assess the severity of the following PTSD symptom clusters: avoidance behaviors, intrusion symptoms, negative alterations in mood and cognition, increased arousal behaviors, and trauma-related sleep disruptions. Cronbach’s alpha coefficient in our sample was 0.94.

Additionally, exposure to potentially traumatic events was measured by the traumatic Life Events Checklist (LEC) [76]. THE LEC is a 17-item self-report measure in which participants are asked to rate each item with 6 response options: happened to me, witnessed it, learned about it, part of my job, not sure, or does not apply. Higher scores indicated greater degrees of exposure to potentially traumatic events. Previous versions of the LEC have shown validity and reliability across various samples [76]. Cronbach’s alpha coefficient in our sample was 0.78.

#### 2.3.4. Statistics

To test hypotheses 1a–c, we used independent samples *t*-tests to compare groups (meditators vs. controls). Pearson correlations were used for hypotheses 2a–c. The inference criteria for determining significant effects were the standard *p* < 0.05, where confirmatory and exploratory hypotheses were addressed as 2-tailed.

For all the behavioral tasks, we set a threshold for excluding outliers of three standard deviations. Thus, in the emotion recognition test (ERT), one meditator and one control participant were excluded for extreme values. For the implicit association test (IAT) and compassion (SoVT), one control participant was excluded.

### 2.4. Neural Data

#### 2.4.1. MEG Data Processing and Construction of Neural Measures

MEG data were processed in alignment with Trautwein et al. [67] using Matlab (R2020b, MathWorks Inc., Natick, MA, USA) and FieldTrip toolbox version number 20201205 [77]. Artifacts caused by the power line frequency, building vibrations, and heartbeats were cleaned by recording on extra channels: recordings from accelerometers attached to the MEG gantry; ECG estimates from the MEG average using an algorithm described in the following website: https://github.com/yuval-harpaz/cleanMEG_BIU, accessed on 30 August 2021). The data from the 2 meditative conditions and resting state were then segmented into 1 s epochs with 250 ms overlap between adjacent epochs (resulting in 1189 epochs). Then, a blinding procedure was implemented by randomly shuffling epochs and removing condition labels to ensure rigor and non-biased data processing. Afterward, each epoch was visually examined for muscle and jump artifacts. Contaminated epochs were discarded, and for all datasets, two consistently noisy channels were removed, while for one subject, one noisy channel was removed and then replaced by interpolation.

Independent component analysis (ICA) was applied to remove eye movement and remaining heartbeat artifacts. Segmented data were downsampled to 300 Hz to speed up data decomposition. The data were then decomposed into a set of independent components (246, as the number of sensors after removal of 2 noisy sensors) ordered by the degree of their explained variance. Components indicating heartbeats or eye movements, determined from a visual inspection of the 2D scalp maps and time course of each component, were removed. The remaining components were then used to reconstruct the pre-down-sampled data.

#### 2.4.2. Sensor-Level Analyses

The cleaned data were then unblinded, and epochs were multiplied by a Hanning taper and subjected to a Fast Fourier Transformation (FFT) for the frequencies ranging from 1 to 90 Hz at 1 Hz resolution. Data were averaged across all trials, and power values were normalized by dividing each value by the mean power across the overall spectrum. To test effects on the sensor level, clusters were formed in sensor and frequency space, delimited by the range of prespecified frequencies of interest (14–33Hz), and subjected to non-parametric cluster-based randomization tests to evaluate the significance of differences between conditions. To avoid the potentially biased selection of tested sensors and/or frequencies, the cluster-based test computes Monte Carlo estimates of significance probabilities from the permutation distribution by evaluating the extent and intensity of adjacent effects within the overall space. Specifically, clusters were formed by thresholding individual values at *p* = 0.01 and then evaluated based on the maximum sum of t-values within a cluster. For the randomization distribution, 10,000 permutations or (when lower) all possible permutations were drawn. Hypotheses were then evaluated at a two-tailed alpha threshold of *p* = 0.05.

#### 2.4.3. Source-Space Projection

To localize cerebral sources of the effects, spectral power at the peak frequency of the sensor level test (27 Hz) was transformed to source-level power estimates using adaptive spatial filtering (beamforming) relying on partial canonical correlations. This approach solves the inverse problem of relating sensor-level measurements to their neural sources by constructing spatial filters that optimally pass activity from locations of interest while suppressing activity that is not of interest. For each participant, a semi-realistic head model of volume conduction was built based on a single-subject MRI template that was linearly mapped to fit each participant’s digitized head shape. A regular 3-dimensional source grid (1 cm resolution) was then inversely warped to fit the participant’s head model. This procedure facilitates the group analysis because no spatial interpolation of the volumes of reconstructed activity is required. For each grid position, a lead field matrix was calculated according to the head position in the system and the volume conduction model. We computed the cross-spectral density matrix between all MEG sensor pairs from the Fourier transforms of the tapered data epochs. Based on the lead field and cross-spectral density matrices, a common spatial filter for all the conditions was then constructed for each grid point. Single-trial source power estimates were then calculated based on the common spatial filter and averaged across each condition. To evaluate the significance of differences between conditions, we used non-parametric cluster-based randomization tests, as well as to avoid biased selection of tested voxels with similar parameters for thresholding clusters (the same as described for the sensor-level analyses).

## 3. Results

### 3.1. Group Differences Between Meditators and Controls in Social Processing

**Figure 2:**
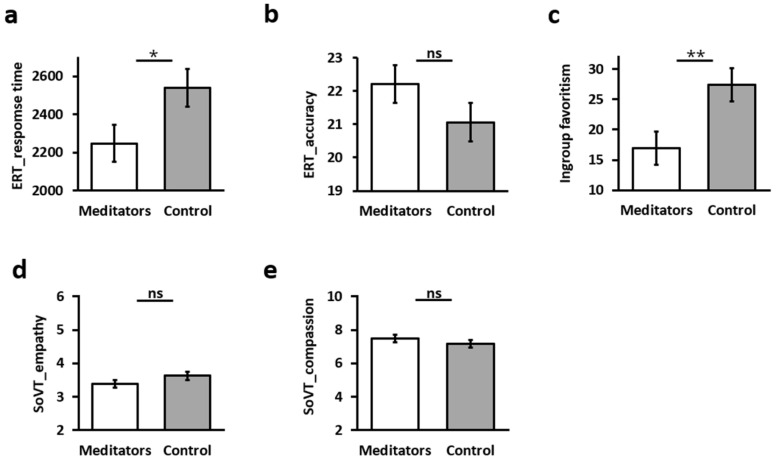
Results of behavioral tasks comparing between meditators and controls using *t*-test: (**a**) Emotion recognition test (ERT) response time. (**b**) Emotion recognition test (ERT) accuracy. (**c**) Ingroup favoritism measured by the implicit association test (IAT). (**d**) Socio-affective video task (SoVT) empathy. (**e**) Compassion. Error bars represent standard error of the mean; * *p* < 0.05 ** *p* < 0.01; ns: non-significant difference.

To test the preregistered hypotheses 1a–c, we compared meditators and controls in the behavioral tasks by employing an independent-sample two-tailed *t*-test (see Table 1 and Figure 2). To evaluate the normality of the data, a Kolmogorov–Smirnov test was conducted for all behavioral measures. The results indicate that the distribution of all behavioral measures did not significantly deviate from normality for both groups: meditators (ranging from D(42) = [0.06–0.12], *p* = [0.10–0.20]) and controls (ranging from D(53) = [0.08–0.13], *p* = [0.10–0.20]). Thus, the data were deemed suitable for parametric testing. To verify the assumption of homogeneity of variances, Levene’s test was conducted. Levene’s test indicated no significant differences in variances among the groups for ERT response time (F = 1.565, *p* = 0.214), accuracy (F = 0.121, *p* = 0.729), IAT (F = 1.515, *p* = 0.222), SoVT empathy (F = 0.002, *p* = 0.967), and compassion (F = 0.281, *p* = 0.597), suggesting that the assumption of equal variances was met.

For the emotion recognition task (ERT), meditators showed faster emotion recognition (lower response time, Figure 2a) and higher composite scores (Figure 2b), as hypothesized, but did not differ significantly from controls in the accuracy scores. For the implicit association test (IAT), meditators had significantly lower ingroup favoritism scores (Figure 2c), as hypothesized, indicating reduced prejudices towards outgroup members. Lastly, both groups did not differ in the socio-affective video task (SoVT) on both the measures of empathy and compassion (Figure 2d,e).

A follow-up analysis for the emotion recognition test (ERT) was performed to rule out a speed–accuracy trade-off in which meditators were faster than controls at the expense of accuracy, especially in one affect (positive or negative emotions). The findings showed that meditators were significantly faster than controls across all emotions (all r values > −2.044, all *p*-values < 0.05) except “surprise” (*t*(94) = −0.867), *p* = 0.388). Regarding accuracy, there was only one emotion that differed significantly between meditators and controls: “happy” (*t*(95) = 2.499), *p* = 0.014), in which meditators were faster.

### 3.2. Extracting the Neural SB Flexibility Indices

**Figure 3:**
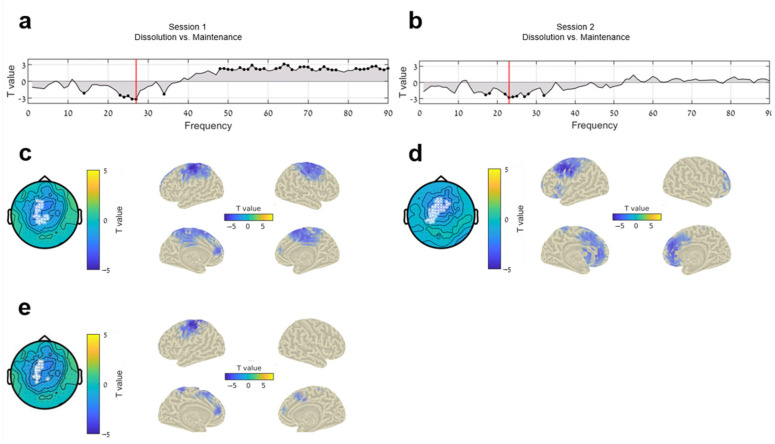
Long-term change effects from session 1 and 2: (**a**,**b**) Line plots of power spectra in all relevant frequencies (1–90 Hz) between conditions (SB dissolution vs. SB maintenance) for Session 1 and 2 (respectively). Black dots highlight frequency bins with significant differences between conditions (uncorrected), and red lines denote the peaks in power spectra. (**c**,**d**) Left panels show scalp topographies with cluster test results in sensor-frequency space for the peak frequency; right panels show source estimates for sessions 1 and 2 (respectively). (**e**) Left and right panels show the overlap in scalp topographies and source (respectively) of sessions 1 and 2, from which the neural index (SBn) was extracted.

We first compared the neural SB dissolution effects measured in the two sessions, the first immediately after the training (SBn in session 1) and the second a year later (SBn in session 2). Power spectra in all relevant frequencies (1–90 Hz) between conditions (SB maintenance vs. SB dissolution) showed negative peaks (high beta) for both session 1 and session 2 (Figure 3a,b). Scalp topographies show the results of cluster tests in sensor-frequency space for the peak frequency (Figure 3c,d, left).

No statistically significant differences were detected between the neural SB flexibility indices of session 1 and session 2. On the sensor level, there were no significant sensor clusters for the difference between both sessions, neither when testing across all frequencies (1–90 Hz), nor averaged across our specific FOI (14–33 Hz) (*t* = 0.129 *p* > 0.05). On the source level as well, no significant voxel clusters emerged when comparing the two sessions. However, the source estimates of SB for session 1 and session 2 were not identical, with session 1 peaking over the somatosensory cortex (Figure 3e) and session 2 peaking over frontal lateral areas (Figure 3d). The SBn flexibility index was thus calculated as the average of the overlapping mask (3e) of both sessions.

**Figure 4:**
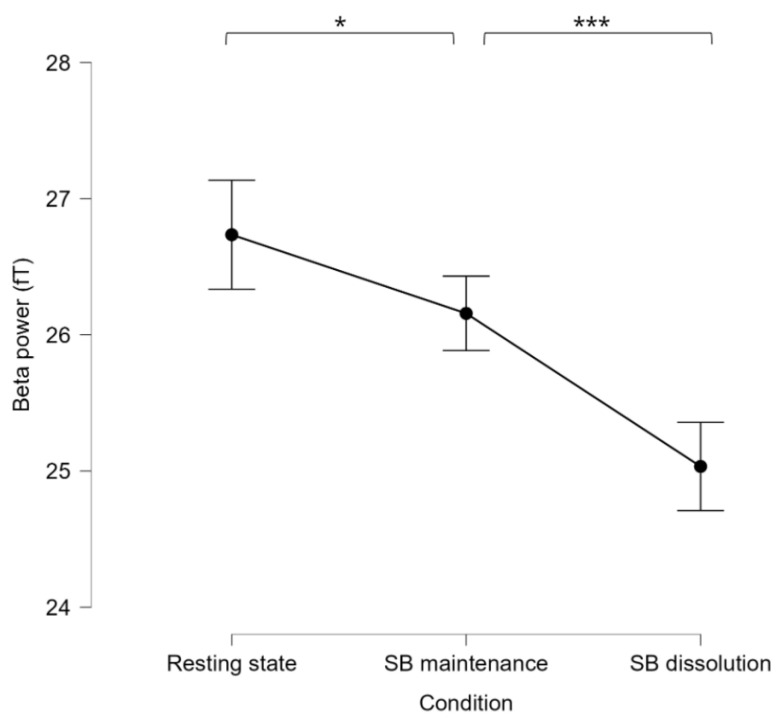
Comparison of beta power estimates (measured by Femtotesla magnetic field, i.e., fT) from the overlapping mask between the meditative states and resting state. Error bars represent standard error of the mean, * *p* < 0.05, *** *p* < 0.001.

A repeated-measures ANOVA determined that mean beta power scores differed significantly between conditions (*F*(2, 64) = 27.469, *p* < 0.001) (Figure 4). The partial eta squared (ηp^2^) was 0.46, suggesting a large effect size. Mauchly’s test indicated that the assumption of sphericity had been violated, χ2(2) = 6.73, *p* = 0.035; thus, the degrees of freedom were corrected using Greenhouse–Geisser estimates of sphericity (ϵ\epsilonϵ = 0.84). The corrected test showed a significant main effect of condition: F(1.67, 53.55) = 29.53, *p* < 0.001. A post hoc pairwise comparison using the LSD correction showed a significant decrease (*p* = 0.023) in beta power between resting state (M = 26.73, SE = 2.16) and SB maintenance (M = 26.16, SE = 2.11) and another significant decrease (*p* < 0.001) from SB maintenance to SB dissolution (M = 25.03, SE = 2.07). These results corroborate our previous results [67] regarding SB flexibility, affirming the link between decreased beta power and volitionally induced self-flexibility.

### 3.3. Ruling Out Associations Between SB Flexibility and Psychopathology

To show that the SB training did not produce psychopathological symptoms, the CDS and anxiety measures were employed twice, pre- and post-training. Using paired-sample two-tailed *t*-tests, our findings indicated a decrease (*t* = 2.41, *p* = 0.020) in depersonalization symptoms between pretraining (M = 17.71, SD = 9.49) and post-training (M = 14.55, SD = 9.86). In a similar manner, a significant decrease in anxiety between the habitual pre-training trait anxiety score and post-training-state anxiety score (collected immediately following the SB alteration task in session 1) indicated a significant (*t*(45) = 4.429, *p* < 0.002) decrease in anxiety between pretraining (M = 37.65, SD = 9.50) and post-training anxiety (M = 30.59, SD = 8.09). These results suggest that the SB training improved the participants’ mental health rather than worsened them.

We also determined that the meditators’ previous post-traumatic experiences did not predict their SB flexibility. The PCL did not predict SBn degree (*r* = −0.08, *p* = 0.675), and the LEC even predicted the opposite: that previous traumatic events predicted less SB flexibility (*r* = −0.37, *p* = 0.043). Finally, no significant associations were found between the SBn and post-training CDS (*r* = −0.16, *p* = 0.358) for the STAI (*r* = −0.07, *p* = 0.683).

### 3.4. Association Between Trait Prosociality and Phenomenological and Neural SB Flexibility Measures (SBp and Neural SBn)

**Figure 5:**
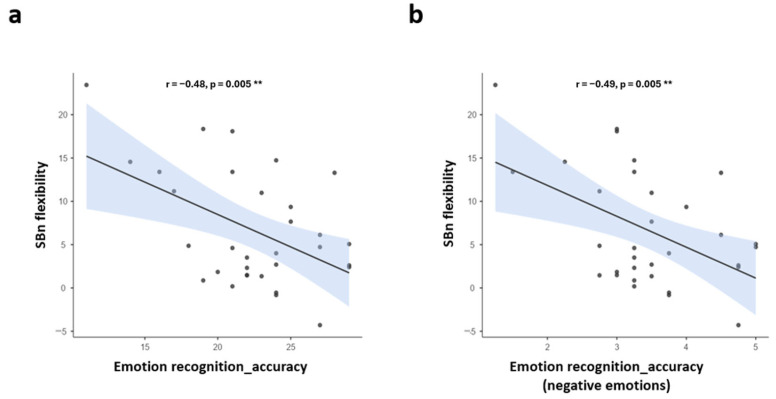
Pearson correlations of self-boundary neural (SBn) flexibility and emotion recognition test (ERT) accuracy: (**a**) Correlation between SBn flexibility and emotion recognition accuracy (across all emotions) and (**b**) correlation between SBn flexibility and emotion recognition accuracy (negative emotions solely), both showing a significant level of ** *p* < 0.01.

To address preregistered hypotheses 2a–c, Pearson correlations between the behavioral measures of prosocial traits and the neural measure of SB flexibility (SBn), as well as phenomenological SB flexibility (SBp), were examined. Contrary to our hypothesis, none of the behavioral measures were associated with SBn or SBp in the direction hypothesized (all *p*-values > 0.1). However, a robust negative correlation (*r* = −0.48, *p* = 0.005) between ERT (accuracy) and SBn was found (n = 32), such that higher flexibility in SBn was associated with less accurate emotion recognition (Figure 5a). To further investigate the nature of this unpredicted but strong negative correlation between SBn flexibility and emotion recognition accuracy, we performed an exploratory analysis in which we dissociated negative (i.e., sad, mad, fear, disgust) from positive (i.e., happy, surprise) emotions. While no correlation was found between SBn flexibility and positive emotions (*r* = −0.21, *p* = 0.239), a negative significant correlation was found with negative emotions (*r* = −0.49, *p* = 0.005; Figure 5b). Thus, higher flexibility was associated with lower recognition of solely negative emotions.

## 4. Discussion

The current study hypothesized that meditators displayed higher prosocial capacities relative to controls and that these capacities were predicted by meditation-induced SB flexibility. Our findings indicated mixed results. Meditators demonstrated significantly faster emotion recognition. Meditators also showed significantly decreased ingroup favoritism, but no significant differences were found for increased empathy and compassion in the SoVT task. Additionally, our hypotheses regarding SB flexibility were not confirmed for the SoVT and IAT tasks and evidenced a significant but opposite correlation than predicted for the emotion recognition task.

The ERT results are aligned with the few studies that directly addressed this aspect of prosociality. Thierry et al. [78] examined the effects of mindfulness on emotion recognition accuracy by implementing a similar behavioral measure to the one used here (following [70]) and comparing students who underwent a mindfulness intervention vs. a control group. Their results demonstrate better post-test emotion recognition for the mindfulness group, indicating the impact of meditative practice on this prosocial trait. In line with this, Creswell et al. [79] measured the link between facets of dispositional mindfulness and emotion recognition accuracy with a computer-based affect labeling task and FMRI. Dispositional mindfulness was correlated with PFC activation and with less connectivity between the PFC and amygdala. By demonstrating less emotional reactivity and more efficient PFC inhibition of the amygdala, the authors suggested that trait mindfulness impacted emotion recognition neural processes.

Similarly, our finding of reduced ingroup favoritism among the meditation group aligns with previous findings that show a general reduction in implicit bias in meditators (for a recent review and meta-analysis, see [80,81], respectively). Mindfulness has been shown to reduce implicit age bias [82] and was associated with implicit affect (i.e., weaker automatic mental associations between “depressed” and “me” concepts) [83]. More directly, Scheps and Walsh [84] reported an association between trait mindfulness and smaller ingroup favoritism, which could be explained by its link to self-boundary constructs, such as “unity with all things” or “boundary diminishment with others and objects”, frequently shown in spiritual contexts [85]. While not assessed in the present study, Lueke and Gibson [86] also found that state mindfulness attenuated ingroup favoritism attenuation. Together, these findings highlight the utility of mindfulness for decreasing bias between ingroup–outgroup and suggest that decreased bias remains a trait.

It is important to note that in contrast to other studies that reported enhanced empathy and compassion in meditators (for a systematic review and meta-analysis, see [8]), no group differences were observed for empathy and compassion in the present study. One explanation for this could be that empathy and compassion were measured here as an affective feeling state using self-report in the SoVT (affective empathy), while in other studies, implicit behavioral measures were used [87,88], such as the Comic Strip Task (CST) paradigm [89], an empathy indicator that is based on how well one can correctly assess other individuals’ mental states (cognitive empathy). In line with this and with a recent review that raised these concerns [8], our explicit measurement of empathy and compassion might have been contaminated by a social desirability bias in the control group [90].

We found no evidence in support of our hypothesis that meditation-induced SB flexibility predicted enhanced prosociality. None of the prosociality measures, targeting different levels of the cognitive hierarchy, correlated with SB flexibility in the hypothesized direction. Thus, it is fair to conclude that meditation-induced flexibility in the SB is not likely to be the mechanism underlying the enhanced prosociality results found among meditators. However, we did find that emotion recognition accuracy showed a highly significant correlation but in the opposite direction to that predicted. Importantly, the results were driven by negative but not positive emotions. In what follows, we attempt to provide an explanation for this result, one that possibly reveals a new understanding of the relation between the SB flexibility phenomenon and the perception of social threats.

As a social organism that depends on its interpersonal connections, a fundamental feature for survival is the need to understand if others are of potential threat (e.g., frown) or benefit (e.g., happy) [91]. Therefore, facial emotion recognition constitutes a fundamental ability upon which more complex types of social cognition can be built, such as the classification of oneself as part of an in- or outgroup, the ability to put oneself in another’s shoes (empathy), and a willingness to help others (based on compassion, or “empathic concern”, see [8]. We suggest that the fact that emotion recognition (and specifically recognizing negative emotions) decreases with increased SB flexibility might be related to a reduced sense of threat, that is, less survival-centered cognition. This is aligned with Deikman’s [92] approach to self-transcendence, which differentiates between two modes of consciousness that comprise two functional categories of self: the “survival self” and the “transcendent self”. The survival self refers to the habitual human state: self-focused and distinct from the environment, characterized emotionally by fear and cognitively by focal attention, sharp perceptual boundaries, logical reasoning, and a sense of linear time streaming from past to future. In contrast, the transcendent self is characterized by resonance with the environment, centeredness upon others, satisfaction and sense of meaning, diffuse attention, blurred perceptual boundaries, and the “now” rather than “linear time” dominating awareness. It is worth mentioning that the meditation-induced altered sense of self is tightly linked to changes in time perception [93]. This probably also plays a role in our study; nevertheless, here, we focus on the self-related aspects. The phenomenological analysis of the SB dissolution state in this cohort [66] indicates a resemblance between this state and the transcendent self (including a wider scope of attention, indistinct and blurred body sensations, expansion beyond body-centered location, and a reduction in the sense of time), thus supporting the proposed reduction in fear, together with diffuse attention, which can explain the reduction observed in negative emotions recognition in this study. This interpretation supports the view of SB as a sort of permeable membrane that mediates the degree of social openness, i.e., the body boundaries mediate between the self and receptivity to the social world [94]; thus, flexible SBs “enhance permeability” and thus reduce threat-centered cognition.

The fact that emotion recognition was the only measure to covary with the neural index of SB flexibility points towards the possibility that the other measurements tap into processes that are higher in the socio-cognitive hierarchy, such as social identity evaluation (measured in the implicit association task) and self-reflective evaluation of one’s own social emotions (measured by SoVT). This is based on one of the distinctions that have been made among task-based measures of social cognition [88,95], separating tasks into lower-level and higher-level social cognition. While lower-level perceptual processes involve involuntary and automatic affect sharing [96], such as recognition of facial expressions (e.g., emotion recognition [97]), the higher-level processes involve integrating and interpreting these cues to infer the mental states of others in a context-sensitive manner [95]. Aligned with this, it has been previously suggested that attempts to uncover brain-behavior relationships are less likely to be successful when dealing with complex behavioral measures that have lower measurement reliability (such as less controlled between-subject measures vs. within-subject lab-controlled measures [98]), which could be argued for psychological measures of open choice as in SOVT vs. reaction times of IAT and ERT. Another possible explanation for the lack of correlation between SB flexibility and IAT relates to our findings of lower ingroup favoritism among meditators: it might be that meditators have already reached a baseline of indifference between in- and outgroup members, i.e., a ceiling effect. This would make the meditator group more homogenous and, therefore, less likely to demonstrate a correlation with SB flexibility.

As part of this study, we also compared the neural SB flexibility measures as acquired in two sessions, approximately one year apart. Our results show no significant differences between the neural SB flexibility in the sessions, thus corroborating our previous study’s [67] results that increasing SB flexibility is characterized by a gradual reduction in high beta. Other studies reported meditation-related reductions in the beta frequency range during different meditation practices compared with rest (for reviews, see [99,100]). The present study further contributes to the literature by showing that these trained SB flexibility capacities are stable and persist over time.

The current findings inform the pattern theory of selflessness (PTSL) [58], which suggests that the self is a dynamic, interconnected pattern of processes, such as embodied, experiential, affective, cognitive, narrative, and social aspects. PTSL posits that meditative practices can reorganize this self-pattern, allowing for experiences of “selflessness”. This model includes six transformative steps. The first three foster long-term changes by integrating a coherent self-concept, cultivating present awareness, and enhancing mindful reflexive awareness. The fourth transformation introduces self-deconstructive states, where awareness becomes non-conceptual and non-dual. The fifth transformation promotes self-pattern flexibility, enabling rapid shifts and integration of various self-aspects. Finally, self-liberation emerges, characterized by a realization of the illusory self, existential resilience, and profound well-being independent of external conditions. The authors term this “selflessness” a trait and consider it a moral characteristic, an attitude that does not prioritize one’s self over others. Our results that self-boundary dissolution is a volitional and persistent learned skill over time provide evidence for the transition from transformation 4 (self-deconstructive states) towards transformation 5, which involves a flexible self-pattern as a trait. It might be suggested that our results showing decreased emotion recognition with increased SB flexibility, which might be related to a reduced sense of threat and less survival-centered cognition, point towards traits described in transformation 6, the moral attitude that does not prioritize one’s self over others.

This study had several limitations: (1) We only collaborated with long-term meditators who underwent a 3-week training and compared them with controls that did not go through a similar procedure but only matched by demographics. (2) We did not track the meditation practice during the one-year period between both sessions, which did not allow us to make conclusions on other trait effect changes in between sessions, except demonstrating a non-significant change in frequency and source. (3) As we used a correlational design, causality between SB flexibility and its prosocial traits cannot be inferred.

## 5. Conclusions

In line with the literature, we were able to show that meditators displayed higher prosocial capacities relative to controls. While our hypotheses regarding the link between SB flexibility and prosociality were not borne out, the surprising finding that emotion recognition is negatively related to meditation-induced SB flexibility led us to a novel conceptualization of the human self-boundaries as a permeable membrane supporting basic features of social processes, as has been suggested by previous studies [47,48] and theoretical accounts [49,50]. This novel and counterintuitive interpretation will require further empirical studies in order to support its validity and in order to examine its possible link to personal implications. Importantly, we also showed that the neural SB flexibility indices were stable over long periods of time; thus, training the capacity to volitionally flex one’s boundaries is one that persists over time and may have important interpersonal implications. Future studies can harness the current frequency- and region-specific findings, as well as utilize new methods of phenomenology interviews [101], to design targeted neurofeedback interventions for promoting SB flexibility in both clinical populations suffering from SB-related psychopathologies as well as in non-clinical populations wishing to experience self-transcendent states.

## Figures and Tables

**Table 1:** Group comparison of behavioral tasks means and standard deviations of controls (n = 52) and meditators (n = 44) in the behavioral tasks. * *p* < 0.05; ** *p* < 0.01.

Task	Measure	Controls Mean ± SD	Mediators Mean ± SD	*t* (df)	*p*	Cohen’s *d*
ERT	Accuracy	21.27 ± 3.94	22.20 ± 4.07	1.42 (94)	0.256	0.23
	Response time	2538.41 ± 709.98	2246.96 ± 649.92	−2.08 * (94)	0.040	−0.43
IAT	Ingroup favoritism	27.46 ± 19.92	16.99 ± 17.65	−2.68 ** (93)	0.009	−0.55
SoVT	Empathy	3.62 ± 1.94	3.38 ± 1.85	−0.59 (86)	0.554	−0.13
	Compassion	7.17 ± 1.45	7.47 ± 1.40	0.60 (87)	0.314	0.21

## Data Availability

The datasets generated and analyzed during this study are available from the corresponding author upon reasonable request, due to ethical reasons. Raw MEG data were cleaned using MATLAB code, which can be found here (https://github.com/yuval-harpaz/cleanMEG_BIU, accessed on 30 August 2021). Further cleaning, preprocessing, and data analyses were conducted via Fieldtrip (https://www.fieldtriptoolbox.org/, accessed on 10 May 2022), which is a publicly available toolbox.
